# Neuroprotection Mediated by Human Blood Plasma in Mouse Hippocampal Slice Cultures and in Oxidatively Stressed Human Neurons

**DOI:** 10.3390/ijms22179567

**Published:** 2021-09-03

**Authors:** Lucia M. Ruiz-Perera, Anna L. Höving, Kazuko E. Schmidt, Sule Cenan, Max Wohllebe, Johannes F. W. Greiner, Christian Kaltschmidt, Matthias Simon, Cornelius Knabbe, Barbara Kaltschmidt

**Affiliations:** 1 Molecular Neurobiology, Faculty of Biology, University of Bielefeld, 33615 Bielefeld, Germany; lucia.ruiz@uni-bielefeld.de (L.M.R.-P.); max.wohllebe@uni-bielefeld.de (M.W.); 2 Institute for Laboratory and Transfusion Medicine, Heart and Diabetes Centre NRW, Ruhr-University Bochum, 32545 Bad Oeynhausen, Germany; k.schmidt10@uni-bielefeld.de (K.E.S.); cknabbe@hdz-nrw.de (C.K.); 3 Department of Cell Biology, Faculty of Biology, University of Bielefeld, 33615 Bielefeld, Germany; sule.evin@yahoo.de (S.C.); Johannes.Greiner@uni-bielefeld.de (J.F.W.G.); C.Kaltschmidt@uni-bielefeld.de (C.K.); 4 Forschungsverbund BioMedizin Bielefeld, OWL (FBMB e.V.), 33617 Bielefeld, Germany; matthias.simon@evkb.de; 5 Department of Neurosurgery, Protestant Hospital of Bethel Foundation, University Medical School OWL at Bielefeld, 33617 Bielefeld, Germany

**Keywords:** human blood plasma, neuroprotection, oxidative stress-induced neuronal death, human serum albumin, human neural crest-derived stem cells

## Abstract

Neuroprotection from oxidative stress is critical during neuronal development and maintenance but also plays a major role in the pathogenesis and potential treatment of various neurological disorders and neurodegenerative diseases. Emerging evidence in the murine system suggests neuroprotective effects of blood plasma on the aged or diseased brain. However, little is known about plasma-mediated effects on human neurons. In the present study, we demonstrate the neuroprotective effect mediated by human plasma and the most abundant plasma–protein human serum albumin against oxidative stress in glutamatergic neurons differentiated from human neural crest-derived inferior turbinate stem cells. We observed a strong neuroprotective effect of human plasma and human serum albumin against oxidative stress-induced neuronal death on the single cell level, similar to the one mediated by tumor necrosis factor alpha. Moreover, we detected neuroprotection of plasma and human serum albumin against kainic acid-induced excitatory stress in ex vivo cultured mouse hippocampal tissue slices. The present study provides deeper insights into plasma-mediated neuroprotection ultimately resulting in the development of novel therapies for a variety of neurological and, in particular, neurodegenerative diseases.

## 1. Introduction

Aging is associated with physical deterioration affecting every organ in the body, being the major risk factor for most neurodegenerative diseases (ND) [1]. For instance, Alzheimer’s disease (AD), Parkinson’s disease (PD), amyotrophic lateral sclerosis (ALS), and vascular dementia occur frequently in the elderly and are therefore strongly linked to aging [2]. Moreover, aging is partly encoded in a blood-based signature, as factors in the circulation such as chemokine C-C motif ligand 2 (CCL2), CCL11, and β2 microglobulin (B2M) have been shown to modulate aging, while other factors including insulin-like growth factor 1 (IGF1), IGF2, or Growth differentiation factor 11 (GDF-11) were reported to rejuvenate several organs including the brain (reviewed in [3]). Regarding the treatment of neurodegenerative diseases, impressive results were obtained by the application of young plasma or serum to murine models for Alzheimer’s disease as well as in normal aging mice [3,4,5]. To translate these results from the murine to the human system, inferior turbinate stem cells (ITSCs) are an interesting model for age-associated neuronal degeneration as they represent a population of neural crest-derived stem cells (NCSCs) in the adult human organism. These cells could be isolated from the respiratory epithelium of the inferior turbinate of the nasal cavity by a minimally invasive surgery and differentiated into cell types of the mesoderm and ectoderm [6]. In previous studies this stem cell population was successfully differentiated into MAP-2^+^/NF200^+^/Synaptophysin^+^/vGlut2^+^ -glutamatergic neurons in vitro and ex vivo and was functionality validated, further demonstrating ITSC-derived neurons as a suitable model for the investigation of neuroprotection [7]. The aging of the brain and neurodegenerative diseases are often associated with oxidative stress [8], which consists of an imbalance between the production and detoxification of reactive oxygen species (ROS) in cells and tissues. As the intrinsic antioxidant defense system of the organism is overwhelmed and the balance is in favor of the highly reactive and unstable oxygen species, the damage of biological molecules occurs. Commonly defined as ROS are superoxide radicals (O_2•_^−^), hydrogen peroxide (H_2_O_2_), hydroxyl radicals (•OH), and singlet oxygen (^1^O_2_) [9]. ROS are involved in cell signaling as second messengers and are produced as a by-product of oxygen metabolism. Further, they are essential for several processes such as protein phosphorylation, apoptosis, immunity, and differentiation [10]. Moreover, the occurrence of oxidative stress is closely associated with aging and age-related disease as ROS cause the characteristic progressive functional loss of organs and tissues. Hence, cardiovascular diseases, chronic obstructive pulmonary disease, chronic kidney disease, neurodegenerative diseases, cancer, and further sarcopenia and frailty are related to oxidative stress [11]. In addition, ROS are frequently produced in the brain, due to its high amount of mitochondria and its elevated oxygen and glucose consumption [12], which makes it on the other hand highly susceptible to ROS-mediated cellular damage via lipid peroxidation during aging [13]. In neurodegenerative diseases such as Alzheimer’s disease, Parkinson’s disease, or cerebral ischemia, ROS can be generated through the excess release of excitatory neurotransmitters such as glutamate. Here, the excitation leads to neuronal damage with excess calcium influx and subsequently to the generation of ROS and reactive nitrogen species (RNS) [14,15]. Kainic acid (KA) (2-carboxy-4-isopropenyl-pyrrolidin-3-ylacetic acid) is a nondegradable structural analog to glutamate and therefore acts as a neurotoxic drug that binds to the α-amino-3-hydroxy-5-methyl-4-isoxazolepropionic acid (AMPA) and KA receptors in the brain [16]. The neurotoxicity of KA is considered to be 30-fold higher than that of glutamate [17], leading to an increase of intracellular ROS followed by neuronal death [18,19,20]. For instance, systemic administration of KA to rats led to the cell death of AMPA and KA receptor-equipped pyramidal neurons in the cornu ammonis 1 (CA1) and cornu ammonis 3 (CA3) regions of the hippocampus, while granule cells of the dentate gyrus (DG) were resistant [21]. Since excitatory stress and the resulting intracellular ROS are considered important drivers of neurological aging [22,23], KA-induced hippocampal damage in rodents is a well-known model for human aging and neurodegenerative disorders [23,24,25]. To counteract the damage caused by ROS, cells possess an antioxidant defense system which can be distinguished by enzymatic and non-enzymatic antioxidants [10]. For example, the glycosaminoglycan hyaluronic acid (HA), which is one of the main components of the brain extracellular matrix, was described to have a great antioxidant activity [26] as well as anti-inflammatory properties [27]. Human serum albumin (HSA), which accounts for 55% of proteins present in the human plasma proteome and therefore is the most abundant protein in human plasma, further possesses a high anti-oxidative activity [28]. Accordingly, a low antioxidant capacity of plasma has been linked to ischemia and neurological impairments in stroke, suggesting plasma as a mediator for neuroprotection [29,30]. In vivo studies confirmed the neuroprotective effect of fresh frozen plasma (FFP) after hemorrhagic shock (HS) and traumatic brain injury (TBI) by significantly reducing lesion size and swelling in the porcine system [31,32].

Extending these promising observations, we investigated the effects of human plasma on oxidatively stressed ITSC-derived human neurons as well as on ex vivo mouse hippocampal slice cultures in the present study. Notably, we detected strong plasma-mediated neuroprotection in both experimental systems. Moreover, aiming to reduce the number of active plasma components, we further applied HSA as the most abundant plasma protein and detected similar neuroprotection in human neurons as well as in the mouse hippocampus ex vivo. We thus show for the first time the antioxidative effects of human plasma and its components in human neurons on the single cell level. Our observations may enable a future determination of the underlying molecular pathways driven by plasma and ameliorate plasma-based therapies against neurodegenerative diseases.

## 2. Results

### 2.1. Human Plasma Protects Ex-Vivo Cultured Mouse Hippocampal Slices from Excitatory Stress

**Figure 1:**
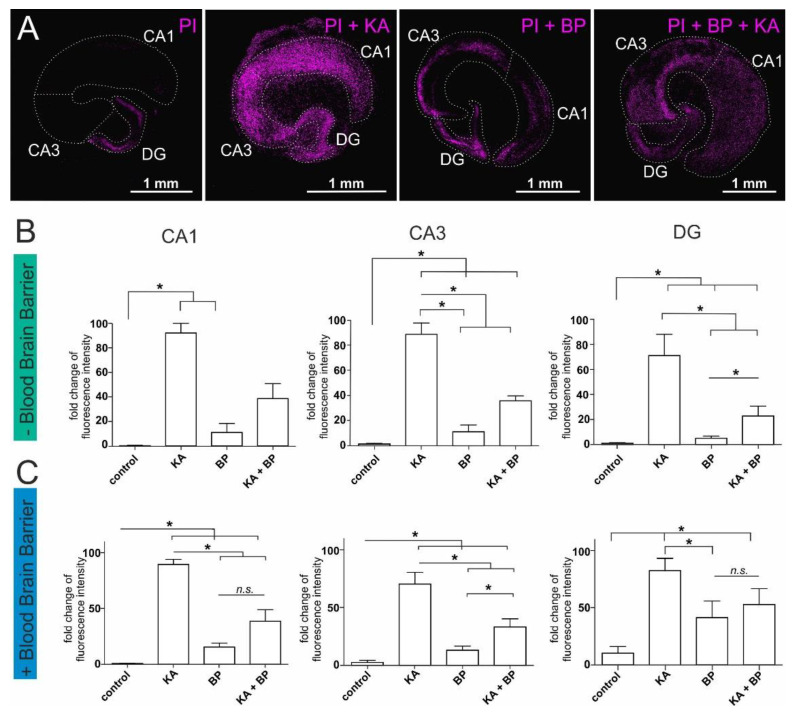
Blood plasma-mediated protection against excitatory stress applied on organotypic mouse hippocampus slice cultures. Treatment of hippocampal slice cultures with kainic acid (KA) results in cell death which can be measured by staining with propidium iodide (PI) (purple color). (**A**) Exemplary pictures of hippocampi in the respective treatment conditions with PI alone as untreated control, PI+KA, PI+ blood plasma (BP), and PI+KA+BP. For better visibility, the fluorescence intensity of these exemplary pictures was amplified. The hippocampal regions are encircled with a dotted line. More unmodified exemplary pictures of PI-stained hippocampal slices can be found in the supplementary material (Supplemental Figure S1). (**B**) Cell death measurements within the different areas of interest in the hippocampus: in the cornu ammonis region 1 (CA1), the cornu ammonis region 3 (CA3), and the Dentate gyrus (DG) of hippocampal slice cultures without blood-brain barrier. (**C**) Cell death measurements in the CA1, the CA3, and the DG of hippocampal slice cultures on top of an artificial blood-brain barrier. Each experiment was performed using 3–4 biological replicates. PI: propidium iodide, KA: kainic acid, BP: Blood plasma, CA1: cornu ammonis region 1, CA3: cornu ammonis region 3, DG: dentate gyrus. * *p* ≤ 0.05 was considered significant. *n.s*. not significant.

Here, we established a system of ex vivo-cultured mouse hippocampi to further investigate the effects of human plasma and HSA within organotypic brain structure. Mouse hippocampal slices are ex vivo cultured tissue compartments suitable for the examination of specific compounds such as KA or plasma. In addition, a layer of cortical endothelial cells served as a model of the blood-brain barrier. This system enabled the possibility to assess the plasma-mediated neuroprotective effects in an organotypic neuronal system under highly defined conditions (Figure 1A, Supplemental Figure S1). The neurotoxic effects of KA were significantly reduced by the application of human plasma in CA1, CA3, and DG. In more detail cell death increased significantly after application of KA in CA1, CA3, and DG compared to untreated control slices. Furthermore, the application of human plasma without KA treatment led to a slight increase in cell death which was not significant compared to the untreated control. Notably, a significant decrease in neuronal cell death was observed upon simultaneous treatment with plasma and KA in CA3 and the DG region but not in CA1 (Figure 1B). Importantly, the neuroprotective effects of plasma were comparable in slices cultured upon a layer of endothelial cells to slice cultures without endothelial cells (Figure 1B,C). As on the one hand a slightly increased cell death in hippocampi treated with plasma, and on the other hand a significant neuroprotective effect of plasma was present, it is suggested that multiple plasma components are active in both processes. We next focused on HSA as only one component of plasma to potentially reduce neurotoxicity and enhance the neuroprotective effect.

### 2.2. Human Serum Albumin Has Neuroprotective Effects on Hippocampal Slice Cultures

**Figure 2:**
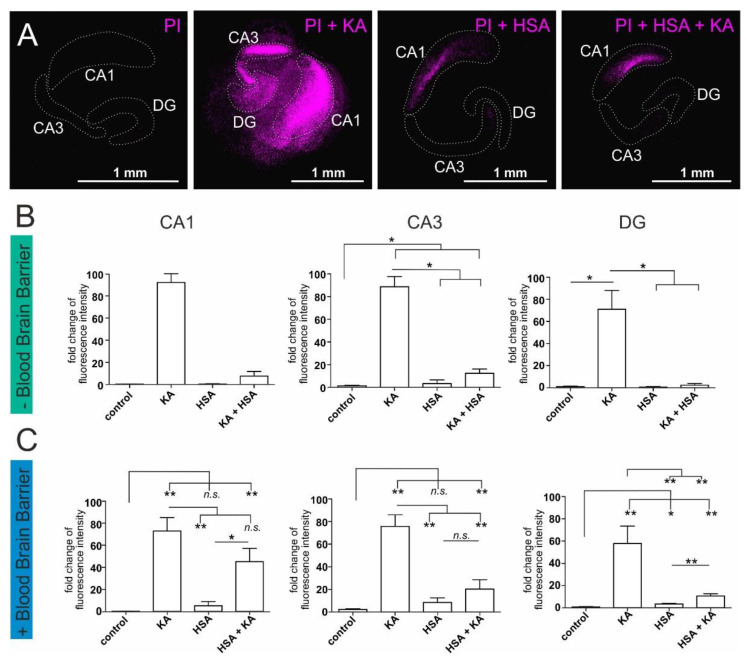
Human serum albumin-mediated protection against excitatory stress applied on organotypic mouse hippocampus slice cultures. Treatment of hippocampal slice cultures with kainic acid (KA) results in cell death which can be measured by staining with propidium iodide (PI) (purple color). (**A**) Exemplary pictures of hippocampi in the respective treatment conditions with PI alone as untreated control, PI+KA, PI+ human serum albumin (HSA), and PI+KA+HSA. For better visibility, the fluorescence intensity of these exemplary pictures was amplified. The hippocampal regions are encircled with a dotted line. More unmodified exemplary pictures of PI-stained hippocampal slices can be found in the supplementary material (Supplemental Figure S1). (**B**) Cell death measurement in the cornu ammonis region 1 (CA1), the cornu ammonis region 3 (CA3), and the Dentate gyrus (DG) of hippocampal slice cultures without blood-brain barrier. (**C**) Cell death measurement in the CA1, the CA3, and the DG of hippocampal slice cultures on top of an artificial blood-brain barrier. Each experiment was performed using 3–4 biological replicates. PI: propidium iodide, KA: kainic acid, HSA: human serum albumin, CA1: cornu ammonis region 1, CA3: cornu ammonis region 3, DG: dentate gyrus. * *p* ≤ 0.05, ** *p* ≤ 0.01 was considered significant. *n.s*. not significant.

HSA is the most abundant protein present in plasma and carries out antioxidant activities [28]. Furthermore, the antioxidant capacity of plasma was shown to mediate neuroprotection in neurological impairments in stroke and ischemia [29]. Here, a neuroprotective effect of HSA, similar to the effect of plasma was observable after the application of HSA to KA-treated hippocampal slices in CA1, CA3, and DG (Figure 2A, Supplemental Figure S1). Again, the treatment with KA alone induced high neuronal death in CA1, CA3, and DG regions while HSA treatment did not result in additional cell death in the same regions in contrast to untreated hippocampi. Interestingly, significant inhibition of the neurotoxic effect of KA was detected in the CA3 and DG regions by treating with HSA likewise to the previously described results (Figure 2B). Furthermore, upon application of a layer of cortical endothelial cells as an artificial blood-brain barrier, a neuroprotective effect of HSA could be identified (Figure 2C). We particularly observed a significant decrease of cell death in the CA3 and DG after treatment with KA+HSA compared to HSA alone (Figure 2C). These observations indicate a central role of HSA in the neuroprotective effect of human plasma in mouse hippocampal slice cultures.

### 2.3. Plasma Mediates Neuroprotection against Oxidative Stress in ITSC-Derived Human Glutamatergic Neurons

**Figure 3:**
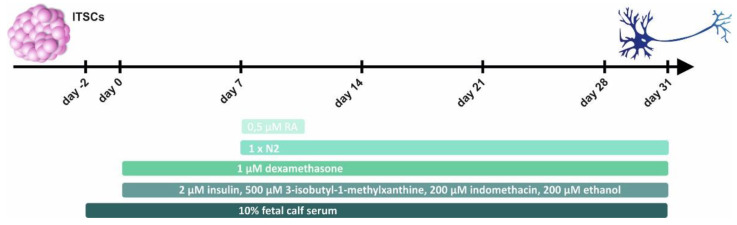
Illustration of the glutamatergic neuronal differentiation from inferior turbinate stem cells (ITSCs). Generation of ITSC-derived neurons for investigation of the neuroprotective effects of human plasma against oxidative stress was achieved by 31 days of neuronal differentiation. ITSCs: inferior turbinate stem cells; RA: retinoic acid.

Next to mouse hippocampal slice cultures, we investigated potential neuroprotective effects of plasma on human neurons differentiated from ITSCs according to our established protocol (Figure 3) [7,33].

**Figure 4:**
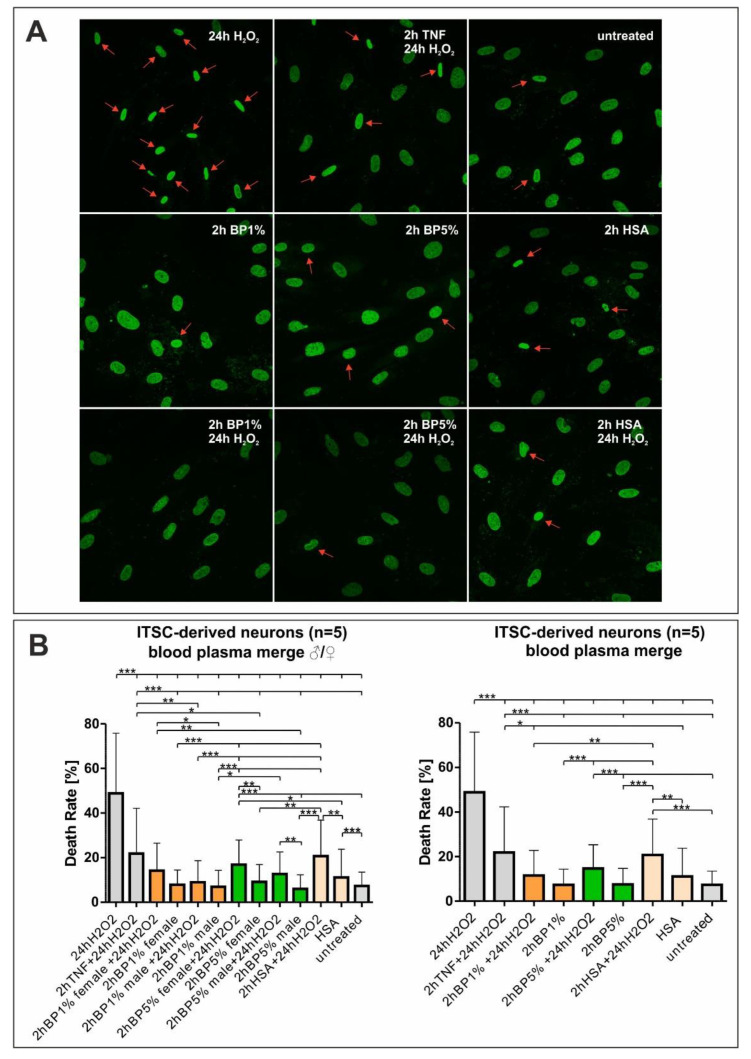
Human plasma mediates neuroprotection against oxidative stress (**A**) Nuclear counterstaining with 4′,6-diamidino-2-phenylindole (DAPI) (green color) showed the morphological changes in nuclear shape, indicated by strongly condensed or fragmented chromatin, after the different treatments revealing an increase in cell death. Fragmented or condensed nuclei are indicated by a red arrow. (**B**) For analysis of human neuronal survival, the amount of nonviable inferior turbinate stem cell (ITSC)-derived neurons recognized by nuclear condensation and/or fragmented chromatin was counted by randomized analysis of 5 pictures per treatment, and donor and death rate was calculated. Quantification of neuronal death for the data collected from three male plasma and two female plasma donors depicted no sex specificity, but an overall neuroprotective effect against oxidative stress treatment. ITSCs: inferior turbinate stem cells; TNF: tumor necrosis factor α; BP: blood plasma; HSA: human serum albumin, H_2_O_2_: hydrogen peroxide. * *p* ≤ 0.05, ** *p* ≤ 0.01, *** *p* ≤ 0.001 was considered significant.

**Figure 5:**
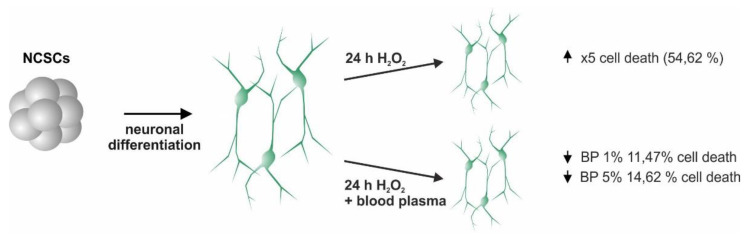
Plasma reduces cell death of inferior turbinate stem cell (ITSC)-derived neurons upon oxidative stress treatment. Application of 1% plasma reduces cell death to 11.47% as the application of 5% plasma reduces cell death to 14.62% (indicated by arrows). Oxidative stress treatment with H_2_O_2_ leads to an increased death rate of 54.62% (indicated by arrow). Scheme summarizes the data shown in Figure 4. BP: blood plasma, H_2_O_2_: hydrogen peroxide.

In a previous study, we already reported a neuroprotective effect on oxidatively stressed ITSC-derived neurons, which was mediated by the tumor necrosis factor alpha (TNF-α) [7]. Extending our previous findings and the observations shown above, ITSC-derived glutamatergic neurons were investigated in an experimental approach testing the neuroprotective effects of human plasma in comparison to HSA and TNF-α with respect to oxidative stress. Therefore, inferior turbinate stem cells were differentiated into glutamatergic neurons and treated with 1% or 5% plasma from male and female donors. Additionally, H_2_O_2_ was applied to induce oxidative stress in the differentiated neurons. After nuclear counterstaining, five pictures per condition were taken and analyzed to evaluate the death rates. Dead cells were characterized by deformed and shrunken nuclei (Figure 4B). Interestingly, intrapopulational differences were visible in the cellular response to H_2_O_2_, where single cells entered apoptosis while neighboring cells were not affected by the H_2_O_2_-stimulus. Untreated control cells showed a death rate of approximately 10% (Figure 4A). ITSC-derived neurons treated with H_2_O_2_ for 24 h showed a death rate of 54.63%, while neurons pre-treated with TNF-α for two hours followed by the addition of H_2_O_2_ for 24 h showed a significantly reduced death rate of 21.8% (Figure 4A). Neurons, treated with 1% blood plasma (BP), 5% BP, or HSA only showed cell death comparable to the untreated control. Strikingly, pre-treatment prior to oxidative stress insult with either 1% or 5% plasma from female and male donors significantly reduced cell death below 20%. However, no significant differences in the neuroprotective effect were apparent between the applied plasma concentrations or the sexes of the plasma donors. Further, pre-treatment with HSA, the most abundant protein in plasma, also reduced cell death to 20% (Figure 4A and Figure 5). Interestingly, cell death in neurons treated with 1% BP and H_2_O_2_ seemed to be slightly but significantly decreased compared to TNF-α + H_2_O_2_ or HSA + H_2_O_2_, suggesting neuroprotective activities of plasma beyond the antioxidative effect of its most abundant protein HSA.

## 3. Discussion

The aging phenotype is defined as progressive loss of physiological integrity leading to impaired function and increased vulnerability to death [34,35]. Notably, the accumulation of intracellular ROS contributes to the aging phenotype and is strongly linked to age-related diseases which comprise neurodegenerative and cardiovascular diseases. For instance, Alzheimer’s disease and Parkinson’s disease occur frequently in the elderly [35,36,37]. Interestingly, circulating factors such as the hormones (IGF1, IGF2) or cytokines like GDF-11 are suggested to mediate the aging process as previous studies confirmed a rejuvenating effect of whole blood from young mice on tissues and organs of old mice by performing parabiosis experiments (reviewed in [3]). This effect was shown for tissues of muscle, liver, brain, spinal cord, olfactory system, and heart. In addition, fresh frozen plasma (FFP) could significantly reduce lesion size and swelling after hemorrhagic shock and traumatic brain injury in the porcine system [31,32]. However, plasma-based protection against neurodegenerative processes or even a rejuvenation of aging organs could not be reproduced in the human system so far.

In general, human plasma is known to carry out an antioxidant activity (AOA) and is crucial for the ROS defense system of the body. For instance, in a previous study, we reported the oxidative stress response pathway (P0046) to be the second most enriched GO term in RNA sequencing data of human cardiac stem cells (hCSCs) treated with human blood serum. Application of the Kyoto Encyclopedia of Genes and Genomes (KEGG) pathway analysis to the same data set demonstrated the upregulation of the glutathione metabolism (hsa00480) and indicates a possible antioxidative effect of blood serum on hCSCs [38]. In addition, several diseases, such as ischemia and neurological impairment in stroke have been linked to a low antioxidant capacity of plasma, which is therefore suggested to be neuroprotective to oxidative stress [29,30,39]. In neurodegenerative diseases (ND), the excitatory release of neurotransmitters such as glutamate leads to excess calcium influx and subsequently to the generation of ROS [14,15]. As oxidative stress is strongly linked to the frequent occurrence of NDs in the elderly, it is of great importance to find new methods to counteract ROS-related NDs.

Within this study, we treated ex vivo-cultured mouse organotypic hippocampal slices with human blood serum and its most abundant protein HSA to investigate potential neuroprotective activities against KA-mediated oxidative stress. Since excitatory stress and the resulting intracellular ROS are considered important drivers of neurological aging [22,23], KA-induced hippocampal damage in rodents is a well-known model for human aging and neurodegenerative disorders [23,24,25]. KA-induced hippocampal damage is also widely used to model epilepsy in animal models [40]. Leakage of HSA through the blood-brain barrier is commonly seen in epileptic hippocampi and thought to possibly contribute to epileptogenesis [41]. Notably, in the present study human plasma, as well as HSA, both significantly decreased the amount of neuronal cell death in ex vivo cultivated hippocampal slice cultures. Moreover, after the application of cortical endothelial cells as an artificial blood-brain barrier, the neuroprotective effects of human blood serum and plasma could be determined. These data strongly suggest a central role of HSA in the neuroprotective properties of plasma. The neuroprotection detected here could be at least partially explained by HSA-activity, which is known to possess antioxidant capacities [28], as discussed below. However, the neurotoxicity of KA results from binding to the AMPA and KA receptors leading to excessive calcium-influx and an increase of intracellular ROS [16,18,19,20]. In this case, the detoxification of extracellular ROS by plasma or HSA could not completely explain its neuroprotective effect and strongly suggests the need for a more detailed future analysis of plasma and its neuroprotective components. In addition, adequate cellular model systems are needed to transform these promising results into the human system.

Facing this challenge, primary adult human stem cells are especially suitable for the investigation of age-associated degeneration. Among the distinct adult stem cell populations, neural crest-derived ITSCs from the respiratory epithelium, exhibit an extraordinary broad differentiation potential into mesodermal as well as particularly ectodermal cell types [6]. For instance, ITSCs have successfully been shown to ameliorate the functional outcome in a Parkinsonian rat model [33]. Within this study, ITSC-derived human neurons served as a model system for ROS-mediated neuronal death, which we previously established [7]. Neuronal death was examined in oxidatively stressed ITSC-derived glutamatergic neurons pre-treated with 1% and 5% plasma from male and female donors. Notably, plasma from both, female and male donors, at both concentration levels could significantly reduce oxidative stress-mediated cell death induced by 24 h H_2_O_2_ treatment and therefore strongly suggests a neuroprotective effect of plasma. Interestingly, the medium for ITSC-derived human neurons is supplemented with 10% FCS which does not show a neuroprotective effect against H_2_O_2_. Similarly, FCS was used in a wide range of different studies examining neuroprotection in vitro [7,42,43] without exhibiting an FCS-mediated neuroprotective effect. Thus, neuroprotection of human serum might be a species-dependent effect only observable with human serum or HSA. However, based on our data as well as data from other groups, we are not able to fully explain this phenomenon. From our point of view, the high neuroprotective effects of even 1% human blood plasma in contrast to the totally absent neuroprotection of 10% FCS, underlines its high relevance for future therapeutic approaches. Likewise, HSA was capable of significantly reducing oxidative stress-mediated cell death. HSA carries out a glutathione-linked peroxidase activity and is therefore able to reduce H_2_O_2_ in the presence of a thiol reducing equivalent (e.g., reduced glutathione or dithiothreitol) leading to the production of oxidized glutathione [44]. The possession of the antioxidant activity of HSA may be the reason for these observed effects, although plasma is suggested to contain an even broader range of antioxidant molecules than HSA alone [45,46]. Likewise, our results show significantly elevated neuroprotective effects in H_2_O_2_-treated ITSC-derived neurons upon the application of 1% plasma compared to HSA. Again, these data may indicate additional active components other than HSA, being present in human plasma regulating the neuroprotective response in human neurons. In this regard, the plasma components alpha-1-proteinase inhibitor and inter-alpha-trypsin inhibitor (ITI) are commonly known neuroprotective proteins in the CNS [47,48], which particularly protect HA from depolymerisation, in turn, scavenging ROS. As a further component of plasma, the tissue inhibitor of metalloproteinase 2 (TIMP2) has been shown to increase synaptic plasticity in the murine hippocampus although the authors did not investigate a potential direct neuroprotective effect [4]. However, the murine system as a model for human aging and disease needs to be taken into account carefully regarding the differences and similarities between the species [49]. Moreover, the complexity of the diverse neurodegenerative diseases additionally increases the number of potentially neuroprotective components carried by human plasma as well as their combinations. For instance, in a previous study, we demonstrated sex-specific p65-mediated neuroprotection in H_2_O_2_-stressed ITSC-derived neurons [7]. In addition, human stem cells not only present sex-specific differences in their response to diverse stimuli but also intrapopulational differences are often described [50,51,52]. Likewise, our results further show intrapopulational differences in ITSC-derived neurons with neighboring cells being differentially sensitive to oxidative stress.

Finally, our findings may also provide some insight into the cellular basis of certain clinical observations and experiences beyond aging and neurodegeneration. A neuroprotective role for HSA in ischemic stroke (at least in part based on the capacity of HSA for ROS detoxification mentioned above) has already been suggested by preclinical studies in animal models [53]. Unfortunately, a recent clinical trial has failed to translate this work into the clinical setting [54]. HSA has also been investigated as a potential neuroprotective agent in patients with subarachnoid hemorrhage (SAH) caused by ruptured brain aneurysms [55]. The efficacy of triple H therapy (hypertension, hypervolemia, hemodilution) in SAH cases, which develop vasospasm and vasospasm-related stroke following aneurysm hemorrhage, may in part be explained by HSA-mediated neuroprotection. The initial triple H protocols used the colloidal effects of HSA infusions in order to expand the intravasal volume, but it is entirely possible that HSA exerts neuroprotective effects on the cellular level in addition to its role in the optimization of cerebral blood flow [56]. However, volume expansion carries adverse sequelae such as increased brain edema. This latter effect may well dominate over any HSA-mediated neuroprotection which in turn would explain why current infusion protocols in patients with SAH and notably traumatic brain injury no longer include HSA [57].

In summary, our results strongly suggest a neuroprotective effect of plasma on the cellular level, especially against oxidative stress damage. Much of this effect could be probably attributed to HSA, however, we cannot rule out the possibility of other plasma factors contributing to this role. In addition to neurodegenerative diseases, increased levels of ROS, and therefore the occurrence of oxidative stress, have been linked to several other neurological conditions but also aging-related diseases such as cardiovascular diseases, chronic obstructive pulmonary disease, chronic kidney disease, cancer, and sarcopenia and frailty [11]. This underlines the importance to further examine the protective potential plasma harbors with respect to oxidative stress.

## 4. Materials and Methods

### 4.1. Isolation and Cultivation of Human ITSCs

ITSCs were isolated from adult human inferior turbinate tissue obtained by biopsy during routine surgery after informed consent according to local and international guidelines. The cells were expanded within the 3D plasma matrix as described previously [6,58], cultivated in Dulbecco’s modified Eagle’s medium/Ham F-12 (Sigma-Aldrich, St. Louis, MO, USA) supplemented with basic fibroblast growth factor-2 (FGF2; 40 ng/mL; Miltenyi Biotec, Bergisch Gladbach, Germany), epidermal growth factor (EGF; 20 ng/mL; Miltenyi Biotec) and B27 (Gibco) followed by supplementation with 10% of clinically accredited therapeutic human plasma (obtained from Institut für Laboratoriums- und Transfusionsmedizin, Bad Oeynhausen, Germany) and cultivated at 37 °C, 5% O_2_ and 5% CO_2_. All experimental procedures were ethically approved by the ethics board of the medical faculty of the University of Münster (No. 2012–015-f-S).

### 4.2. Glutamatergic Differentiation of Human NCSCs

Cells were differentiated into glutamatergic neurons as previously described [7,33]. ITSCs from three male and two female donors (Table 1) were expanded and dissociated (as described above). Cells were re-suspended in Dulbecco’s modified Eagle’s medium (DMEM) high glucose (Sigma-Aldrich) containing 2 mM L-glutamine (Sigma-Aldrich), penicillin/streptomycin (1×, Sigma-Aldrich), 10% Fetal Calf Serum (FCS; Sigma-Aldrich) and plated at a density of 5 × 10^4^ cells per 24 well plate followed by cultivation at 37 °C, 5% CO_2_ and atmospheric O_2_ in a humidified incubator for 2 days. Cells were further exposed to a neuronal induction medium (NIM) containing 1 µM dexamethasone (Sigma-Aldrich), 2 µM insulin (Sigma-Aldrich), 500 µM 3-isobutyl-1-methylxanthine (Sigma-Aldrich), 200 µM indomethacin (Sigma-Aldrich) and 200 µM ethanol. After 9 days of differentiation, cells were shortly induced with 0.5 µM retinoic acid (Sigma-Aldrich) and 1× N2 supplement (Gibco, Darmstadt, Germany). Afterwards, the medium was changed by removing half of the volume, and addition of fresh pre-warmed NIM containing 1× N2 supplement.

### 4.3. Neuronal Treatments

After 30 days of neuronal differentiation, an oxidative stress assay was performed. For oxidative stress induction, 300 μM of hydrogen peroxide (H_2_O_2_) (Sigma-Aldrich) was applied over 24 h. In order to analyze the neuroprotective role of human blood plasma (BP), differentiated neurons were pre-treated with 1% and 5% of BP in NIM, followed by the H_2_O_2_-treatment. Untreated neurons were used as a control, as well as pre-treated neurons with 1% or 5% human BP alone. In addition, pre-treated neurons with 0.4% HSA alone for 2 h and followed with the H_2_O_2_-treatment were used as a control for the most abundant protein within plasma. Moreover, pre-treated neurons with TNF-α for 2 h alone and followed by H_2_O_2_-treatment were used as a neuroprotection control. For BP pre-treatments, 1% or 5% of BP from six donors (Table 2) were added to NIM, together with 3 IU/mL of heparin to avoid fibrinogen polymerization. Furthermore, HSA was prepared and applied in the same manner as BP5% from a stock solution of 8% and TNF-α was added as a pulse in the NIM (10 ng/mL). Untreated control cells received identical incubation times.

### 4.4. Isolation and Culture of Mouse Endothelial Cells

The isolation of primary mouse endothelial cells was carried out according to Duport and colleagues [59]. Briefly, newborn C57BL/6 wildtype mice (4d) were decapitated, and the cortex was subsequently isolated on an ice-cold surface. The tissue was chopped and digested in 0.1% Collagenase (Worthington, Lakewood, NJ, USA) at 4 °C overnight. To isolate endothelial cells from the suspension, a centrifugation step with Percoll (VWR, Darmstadt, Germany) followed. Endothelial cells were counted and seeded in collagen-coated cell culture dishes (Sarstedt, Nümbrecht, Germany) for further expansion in an endothelial cell medium. For an artificial blood-brain barrier, endothelial cells were seeded on collagen-coated membranous cell culture inserts for 6-well-plates (Millipore, Burlington, MA, USA) at a density of 1 × 10^5^ cells/well.

### 4.5. Ex-Vivo Culture of Mouse Hippocampal Slices

Newborn C57BL/6 wildtype mice (4d) were decapitated, and the hippocampus was subsequently isolated on an ice-cold surface. Using a McIllwain tissue chopper (Mickle Laboratory Engineering, Gomshall, Surrey, UK), each hippocampus was cut in slices of 400 µm and placed on a cell culture insert (Millipore, Burlington, MA, USA) in a 6-well-plate (Sarstedt) in an air-liquid interface with 800 µL of slice culture medium below. Slice culture medium was prepared according to Simoni and coworkers [60]. Slices were quality checked for visibility of the hippocampal structure and then cultures in a humidified incubator at 37 °C with 5% CO_2_. The medium was replaced twice weekly. After 21 days of maturing, the slices were treated with 0.1% propidium iodide (PI) (Sigma Aldrich) for 2 h followed by 0.01% PI, 10% human plasma or 0.8% HSA (Sigma Aldrich), 3 U/mL heparin (Sigma Aldrich) and 500 µM KA (Tocris Bioscience, Bristol, United Kingdom) for an additional 16 h. Slices were fixed in 4% paraformaldehyde (Carl Roth GmbH, Karlsruhe, Germany) for 1 h and the membrane was mounted on a glass slide in Mowiol-4-88 (Carl Roth). Fluorescence imaging was performed using confocal laser scanning microscopy (LSM 780; Carl Zeiss, Jena, Germany).

### 4.6. Staining of Human Nuclei

Differentiated NCSCs were fixed in phosphate-buffered 4% paraformaldehyde (pH 7.4) for 15 min at room temperature (RT) followed by 3 wash steps in phosphate-buffered saline (1× PBS). Cells were permeabilized with 0.02% Triton X-100 for 30 min at RT, followed by nuclear counterstaining with 49,6-diamidino-2-phenylindole (DAPI; 1 µg/mL; Sigma-Aldrich) for 15 min at RT. After 2 washing steps with PBS, cells were rinsed with H_2_O and immediately mounted in Mowiol-4-88. Fluorescence imaging was performed using confocal laser scanning microscopy (LSM 780; Carl Zeiss, Jena, Germany) and analyzed using ZEN software (version 2011 SP7; use time 2019–2020) from the same provider or ImageJ.

### 4.7. Image Analyses and Quantification

Image acquisition settings were kept constant across treatments and imaging was randomized. To detect neuronal death, we analyzed nuclear chromatin morphology using DAPI staining in Images from a 40× Objective, recognizing the nonviable neurons by nuclear condensation and fragmented chromatin. For analysis of human neuronal survival, the amount of nonviable ITSC-derived neurons recognized by nuclear condensation and/or fragmented chromatin was counted, and the death rate was calculated for all NCSCs donors investigated (Table 1), by analyzing 5 pictures per treatment and the donor as previously described [7] using the formula (number of dead cells/total number of cells) × 100. To avoid analysis-based bias, the counting of the condensed nuclei was randomized. Examples of fragmented or condensed nuclei are marked in Figure 4.

To assess cell survival in images of mouse hippocampal slices, the mean fluorescence intensities of 3–4 biological replicates for each treatment were measured in the respective regions CA1, CA3, and dentate gyrus using the ImageJ software.

### 4.8. Statistical Analyses

Statistical analysis was performed using Past3 and GraphPad Prism 5 (GraphPad Software (version 5.0; use time 2019–2020), La Jolla, CA, USA). Normality was refuted using the Shapiro-Wilk normality test. Homogeneity of variance was tested using Levene’s test and the non-parametric Kruskal-Wallis test was applied to compare medians between different donors (*** *p* value < 0.001). Tukey’s post hoc test served to identify the significance of the differences between the groups, by comparing the medians (* *p* ≤ 0.05, ** *p* ≤ 0.01, *** *p* ≤ 0.001).

## Figures and Tables

**Table 1:** ITSC donors.

Donor	Sex	Age
K243	**♂**	53
K248	**♂**	45
K246	**♂**	26
K325	**♀**	68
K395	**♀**	61

**Table 2:** Human plasma donors.

Donor	Sex
Ym12	**♂**
Ym15	**♂**
Ym17	**♂**
Yf8	**♀**
Yf7	**♀**
Yf2	**♀**

## Data Availability

Data is contained within the article.

## Supplementary Materials

The following are available online at https://www.mdpi.com/article/10.3390/ijms22179567/s1.
