# Supplementary material for: Neuroprotection Mediated by Human Blood Plasma in Mouse Hippocampal Slice Cultures and in Oxidatively Stressed Human Neurons
Source: Int J Mol Sci. 2021 Sep 3;22(17):9567. doi: 10.3390/ijms22179567 (PMC8430756; doi:10.3390/ijms22179567)
Supplement: Supplementary file 1 [file ijms-22-09567-s001.zip › ijms-1350410-supplementary.pdf]

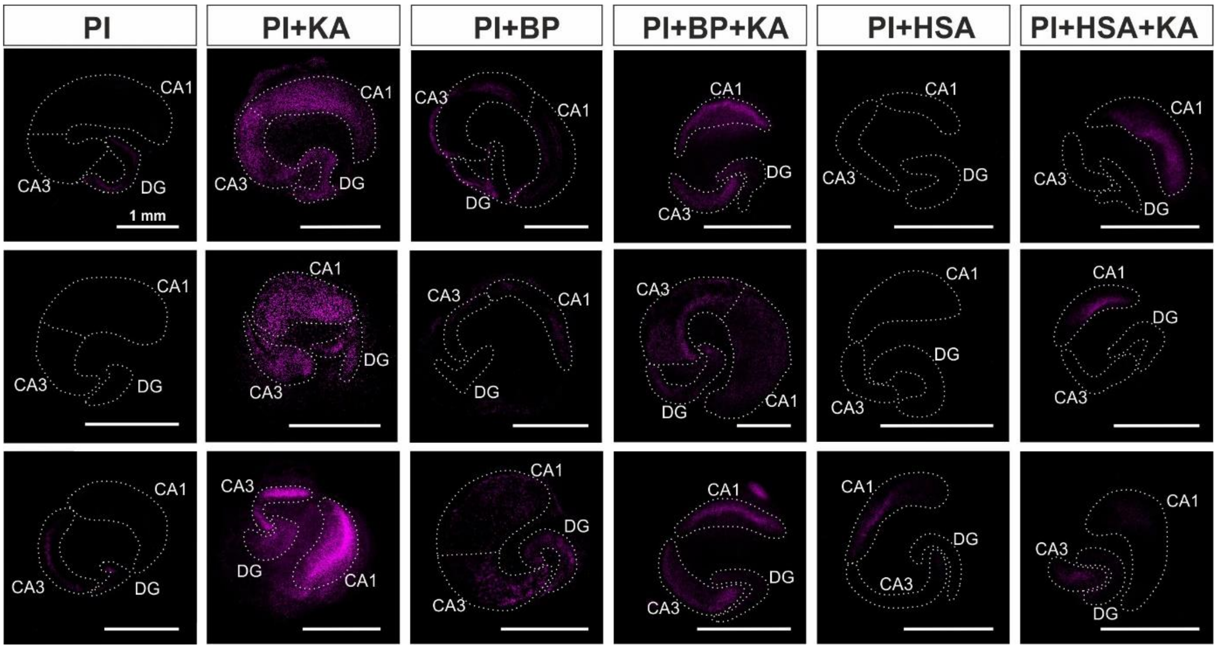

Supplemental Figure S1: Exemplary pictures of PI-stained mouse hippocampal slice cultures. *Treatment of hippocampal slice cultures with kainic acid (KA) results in cell death which can be measured by staining with propidium iodide (PI). PI: propidium iodide, KA: kainic acid, BP: blood plasma, HSA: human serum albumin, CA1: cornu ammonis region 1, CA3: cornu ammonis region 3, DG: dentate gyrus.*
